# Supplementary material for: The causal relationship between the human gut microbiota and pyogenic arthritis: a Mendelian randomization study
Source: Front Cell Infect Microbiol. 2024 Nov 26;14:1452480. doi: 10.3389/fcimb.2024.1452480 (PMC11629706; doi:10.3389/fcimb.2024.1452480)
Supplement: Supplementary file 1 [file DataSheet1.zip › mendelian test/ebi-a-GCST90027713.csv_forest.pdf]

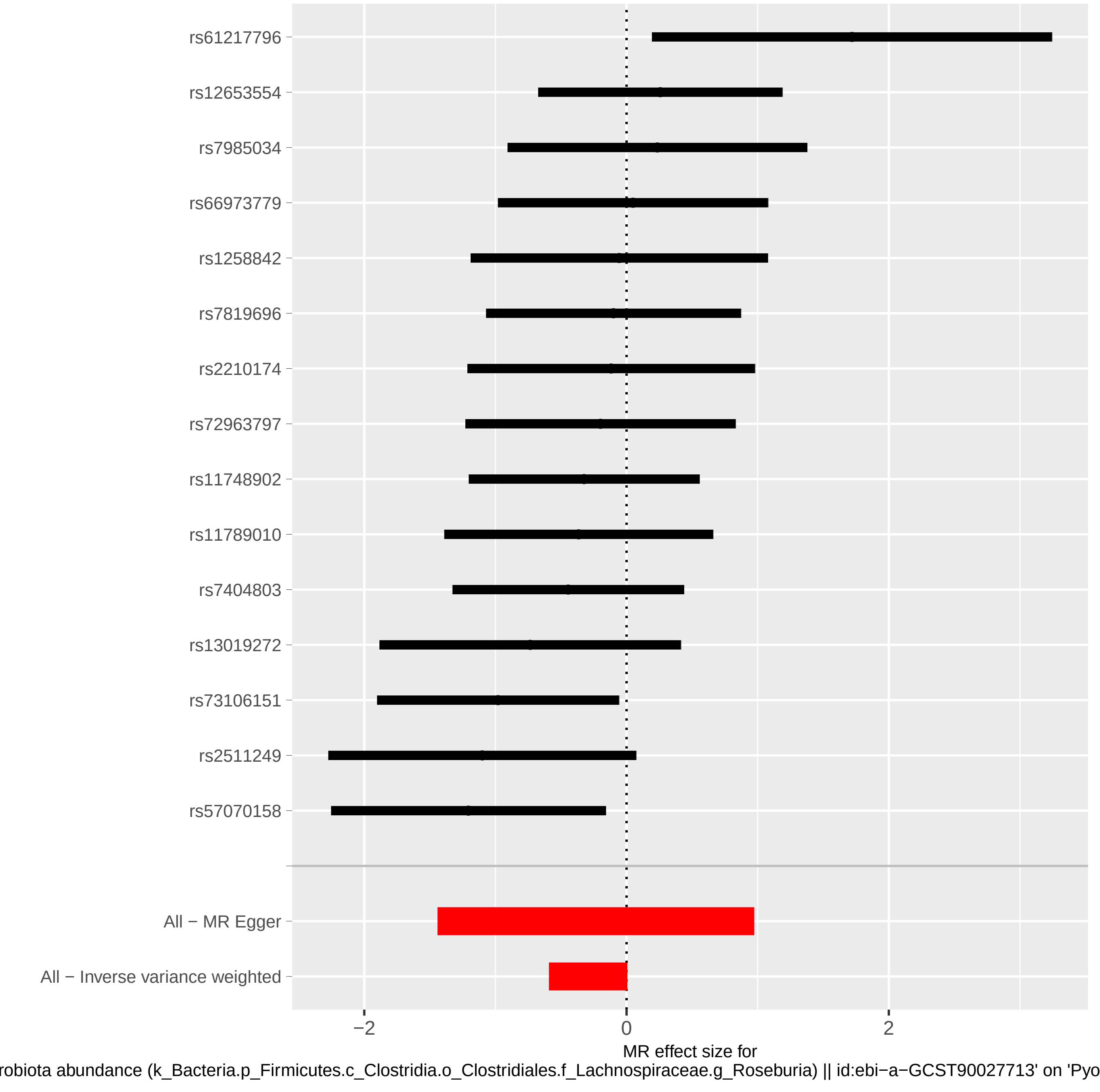
rs61217796
rs12653554
rs7985034
rs66973779
rs1258842
rs7819696
rs2210174
rs72963797
rs11748902
rs11789010
rs7404803
rs13019272
rs73106151
rs2511249
rs57070158
All − MR Egger
All − Inverse variance weighted
−2
0
2
MR effect size for
robiota abundance (k_Bacteria.p_Firmicutes.c_Clostridia.o_Clostridiales.f_Lachnospiraceae.g_Roseburia) || id:ebi−a−GCST90027713' on 'Pyo
